# HATs off to OsHAG704 and OsBPM2: novel regulators of OsRpp30 in rice blast resistance

**DOI:** 10.1093/plphys/kiag268

**Published:** 2026-05-07

**Authors:** Catherine P Freed

**Affiliations:** Department of Biochemistry, University of Wisconsin-Madison, Madison, WI 53706, United States; Assistant Features Editor, Plant Physiology, American Society of Plant Biologists

*Magnaporthe oryzae* is a fungal pathogen that causes rice blast, a devastating disease costing farmers up to 30% in yield losses (Boddy 2016). Rice plants have developed a multi-faceted immune system to combat *M. oryzae* infection. One line of defense involves activating the immune system through protein post-translational modifications (PTMs) (Withers and Dong 2017). Although researchers have begun to explore the impacts of phosphorylation, glycosylation, and ubiquitination on rice blast disease regulation, the role other PTMs, such as acetylation, remain unexplored.

Acetylation is a push–pull relationship between a “writer” histone acetyltransferase (HAT) that donates an acetyl group from acetyl-CoA and an “eraser” histone deacetylase (HDAC) that removes the acetyl group (Eberharter and Becker 2002). Though acetylation is commonly known for its role in modifying histone proteins to regulate gene expression, nonhistone proteins are also acetylated. In fact, nonhistone acetylation of proteins is a strategy used in many plant–pathogen interactions to activate or evade the host immune system (Villette et al. 2025). Interestingly, while HAT-mediated acetylation has not yet been connected to rice immune regulation, past work has shown that HDAC-mediated deacetylation is involved (Li et al. 2021; Yang et al. 2024), opening the door for acetylation as a way to regulate immunity.

A recent study published in *Plant Physiology* by Feng et al. connects HAT-mediated acetylation to rice blast immunity (Feng et al. 2026). The authors previously identified rice RNase P subunit Rpp30 (OsRpp30), a protein involved in tRNA processing and translation that positively regulates rice defense response against pathogens (Li et al. 2021). Their earlier work shows that OsRpp30 was deacetylated by an HDAC and knockout lines of this specific HDAC gene increased plant defense against *M. oryzae*. Given this, the authors hypothesized that acetylation of OsRpp30 positively regulates defense against rice blast disease.

To explore this, Feng et al. examined interactions between OsRpp30 and multiple rice HAT candidates. They found OsHAG704 interacted with OsRpp30 through a series of yeast 2-hybrid, split-luciferase complementation, and co-immunoprecipitation assays. Exploration of previously published transcriptomics data and RT-qPCR analyses also revealed that *OsHAG704* and *OsRpp30* were highly expressed in leaves and moderately expressed in other tissues, suggesting that expression of these genes is coordinated in rice.

To determine whether OsHAG704 is a relevant “writer” for OsRpp30, the Feng et al. transiently expressed OsRpp30 with and without OsHAG704 in rice protoplasts. Quantification of immunoblots showed a significant enrichment in acetylated OsRpp30 proteins when coexpressed with OsHAG704, confirming OsHAG704 acetylates OsRpp30 in vivo. Intriguingly, OsRpp30 protein levels increased when transiently coexpressed with OsHAG704 in rice protoplasts. The authors assessed whether OsHAG704-mediated accumulation of OsRpp30 was dependent on acetylation by exploring the interactions between OsRpp30 and a truncated OsHAG704 protein lacking the acyl transfer domain. Their experiments revealed that OsRpp30 still interacted with and accumulated in the presence of the truncated OsHAG704 protein, suggesting that OsHAG704 stabilizes OsRpp30 through a direct interaction rather than acetylation. Given this interesting result, the authors explored whether the increase in OsRpp30 accumulation was due to increased synthesis or reduced degradation. The authors coexpressed OsRpp30 with OsHAG704 or GFP in rice protoplasts and treated them with cycloheximide, a protein synthesis inhibitor. Their assays revealed OsRpp30 levels decreased significantly slower when coexpressed with OsHAG704, suggesting that OsRpp30 accumulation is increased due to a decrease in degradation.

To explore the relationship between OsHAG704 and OsRpp30 and their impact on defense against rice blast, the authors generated rice transgenic and knockout mutant lines for OsHAG704, referred to as OX-OsHAG704 and *oshag704-1*, respectively. OX-OsHAG704 transgenics inoculated with *M. oryzae* showed significantly enhanced resistance compared to wild-type (WT) plants through reduced leaf lesions and fungal biomass (Fig. 1a and b). Notably, *oshag704-1* mutants showed no significant difference in changes in resistance to *M. oryzae* compared with WT. The authors also reported that transgenic plants overexpressing both OsHAG704 and OsRpp30 had significantly higher resistance to *M. oryzae* inoculations compared to transgenics only expressing OsRpp30.

**Figure 1:**
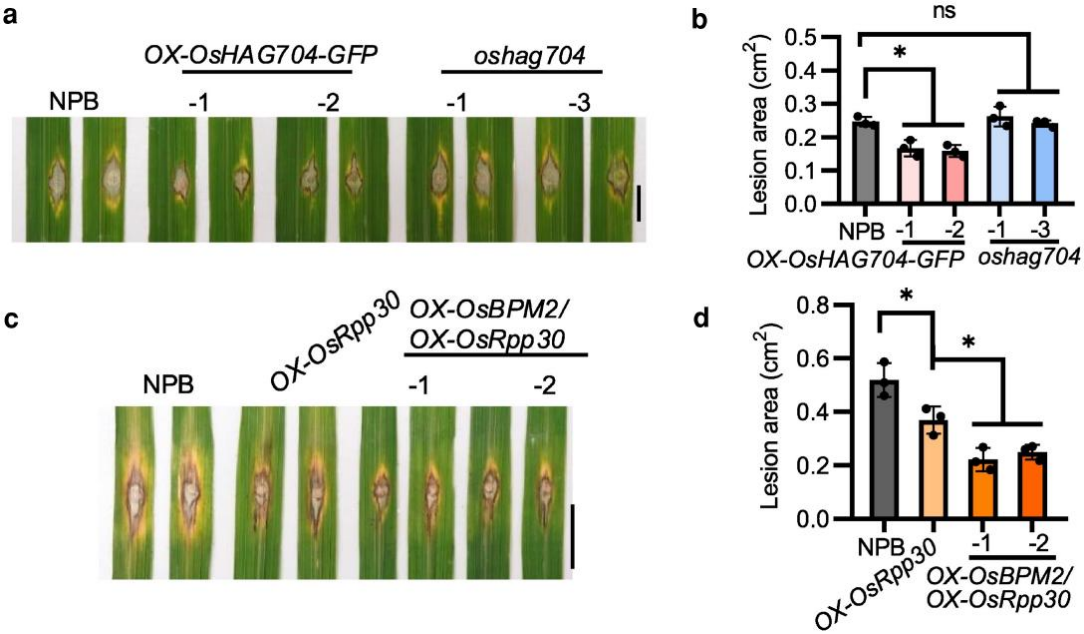
Increased stability of OsRpp30 promotes plant resistance to *M. oryzae*, a fungal pathogen that causes rice blast disease. This figure has been adapted from Figs. 3 and 5 from Feng et al. 2026. a) Disease symptoms of WT (NPB; “Nipponbare”), transgenic (OX-OsHAG704), and knockout mutant (*oshag704-1*) lines for OsHAG704, 12 d postinoculation with *M. oryzae*. Scale bar = 1 cm. b) Quantification of fungal lesions from a). c) Disease symptoms of WT (NPB; “Nipponbare”) and transgenic lines 14 d postinoculation with *M. oryzae*. Scale bar = 1 cm. d) Quantification of fungal lesions from c). Data presented in panels b & d) are represented as means ± SD (n = 3). Statistical differences were determined by Student *t* tests; *P* < 0.05. “ns” indicates not significant.

Feng et al. sought to identify the factors controlling OsRpp30 stability. Through a series of proteasome inhibitor assays and yeast2 hybrid screening of a high-coverage rice E3 ligase library, they identified E3 ligase OsBPM2 as an interactor with OsRpp30. GST pull-down and co-immunoprecipitation assays further showed that OsRpp30 interacts with OsBPM2. Coexpression of OsBPM2 and OsRpp30 also increased accumulation of OsRpp30 protein, a similar result to what was observed with OsHAG704. The fact that OsBPM2, an E3 ligase, stabilizes OsBPM2 was surprising and suggests a less intuitive role for OsBPM2 such as targeting a E3 ligase that degrades OsRpp30 or mediating nonproteolytic ubiquitination in rice. Overexpression of OsBPM2 in stable rice transgenics increased resistance to *M. oryzae* infection, whereas *osbpm2* mutants were more susceptible. Remarkably, OsBPM2 and OsRpp30 had a synergistic effect on enhancing *M. oryzae* resistance as transgenic lines overexpressing both had significantly reduced rates of fungal biomass and infection compared with WT and Ox-OsRpp30 transgenics (Fig. 1c and d).

Since OsHAG704 and OsBPM2 both stabilized OsRpp30, the authors explored the relationship between these 2 proteins. OsHAG704 and OsBPM2 were not found to physically interact based on yeast2hybrid and co-immunoprecipitation assays. Co-infiltration experiments confirmed that OsHAG704 disrupted the interaction between OsBPM2 and OsRpp30. This suggests that OsHAG704 and OsBPM2 have more of a competitive role in regulating OsRpp30 accumulation and stability.

This study explores how increasing OsRpp30 stability in rice promotes resistance to rice blast disease. OsHAG704 and OsBPM2, 2 proteins with distinct biochemical functions, competitively interacted with and boosted OsRpp30 stability in vivo. The broader agricultural implications of this research are exciting as increased OsRpp30 stability enhanced resistance of rice transgenics to *M. oryzae* infection. Further exploration of the regulatory crosstalk between HATs and E3 ligases is critical to enhance our mechanistic understanding of the role PTMs play to improve plant resistance to rice blast disease.

## Data Availability

No new data were generated or analyzed in support of this research.

## References

[kiag268-B1] Boddy L . 2016. Pathogens of autotrophs. The fungi. Academic Press. p. 245–292.

[kiag268-B2] Eberharter A, Becker PB. 2002. Histone acetylation: a switch between repressive and permissive chromatin. EMBO Rep. 3:224–229. 10.1093/embo-reports/kvf053. 11882541 PMC1084017

[kiag268-B3] Feng Q et al 2026. Dual regulation of RNase P subunit Rpp30 by an acetyltransferase and E3 ligase in rice immunity. Plant Physiol. 200:kiag157. 10.1093/plphys/kiag157. 41873718

[kiag268-B4] Li W et al 2021. The rice RNase P protein subunit Rpp30 confers broad-spectrum resistance to fungal and bacterial pathogens. Plant Biotechnol J. 19:1988–1999. 10.1111/pbi.13612. 33932077 PMC8486239

[kiag268-B5] Villette J et al 2025. Insights into the role of lysine acetylation of non-histone proteins in plant immunity. Plant Cell Environ. 10.1111/pce.70139. PMC13551640 40842252

[kiag268-B6] Withers J, Dong X. 2017. Post-translational regulation of plant immunity. Curr Opin Plant Biol. 38:124–132. 10.1016/j.pbi.2017.05.004. 28538164 PMC5644497

[kiag268-B7] Yang Z et al 2024. Histone deacetylase OsHDA706 orchestrates rice broad-spectrum antiviral immunity and is impeded by a viral effector. Cell Rep. 43:113838. 10.1016/j.celrep.2024.113838 38386554

